# Grey System Theory in the Study of Medical Tourism Industry and Its Economic Impact

**DOI:** 10.3390/ijerph17030961

**Published:** 2020-02-04

**Authors:** Hoang-Sa Dang, Thuy-Mai-Trinh Nguyen, Chia-Nan Wang, Jen-Der Day, Thi Minh Han Dang

**Affiliations:** 1 Department of Industrial Engineering and Management, National Kaohsiung University of Science and Technology, No.415, Chien Kung Road, Sanmin District, Kaohsiung City 80778, Taiwan; cn.wang@nkust.edu.tw (C.-N.W.); jdd@nkust.edu.tw (J.-D.D.); 2 La Trobe University, Plenty Rd & Kingsbury Dr, Bundoora VIC 3086, Australia; handtm@gmail.com

**Keywords:** performance evaluation, medical tourism industry, Grey system theory, Asia-Pacific region, Taiwan, sustainable development, economic impact

## Abstract

The Asia-Pacific region is known as a favorite destination for global medical travelers due to its medical expertise, innovative technology, safety, attractive tourism destination and cost advantage in the recent decade. This study contributes to propose an approach which effectively assesses performance of medical tourism industry based on considering the economic impact factors as well as provides a conceptual framework for the industry analysis. Grey system theory is utilized as a major analyzing approach. According to that, factors impact on the sustainable development of medical tourism in Asia-Pacific region could be identified. The performance of each destination in this region was simultaneously revealed. The results presented an overall perspective of the medical tourism industry in the scope of the Asia-Pacific region, and in Taiwan particularly. Data was collected on six major destinations including Singapore, Thailand, India, South Korea, Malaysia and Taiwan. The results proved that tourism sources and healthcare medical infrastructures play a crucial role in promoting the healthcare travel industry, while cost advantage and marketing effectiveness were less considered. In addition, performance analyse indicated that Thailand has a good performance and stands in the top ranking, followed by Malaysia, India, Singapore, South Korea and Taiwan, respectively. The revenue of Taiwan has increased slowly in the last six years, with a market worth approximately NT$20.5 billion, and the number of medical travelers is expected to increase to 777,523 by 2025. The findings of this study are expected to provide useful information for the medical tourism industry and related key players in strategic planning.

## 1. Introduction

The service of healthcare and medical treatment combined with travelling namely “medical tourism” or “healthcare travelling” is a special green industry which has a long history in some developed countries such as Hungary, Belgium, Turkey, and Poland. By and large, medical tourism is believed to be a service which is combined between medical services and tourism activities. Furthermore, it was also defined as travelling activities outside of patients’ home countries to receive medical care [1] or obtain non-emergency medical services [2]. This industry has been rising in the last two decades in the world based on the development of the international commercial activities and special treatment demands abroad. However, along with the rapid development of internet technology improvements in health systems and developments in medical expertise, innovative technology at the world standard of treatment, as well as attractive tourism destinations and cost advantages, the medical tourism industry in developing countries is quickly accelerating. In accordance with, there is a phenomenon of patient mobility, that is a transformation from developed countries to developing countries for treatment [3] and this is expected to bring in lucrative market revenue. Statistical data in 2019 showed that 47 percent of U.S. adults had traveled abroad specifically to receive medical treatment, and that Canada, Mexico, Brazil, China, Singapore, and India are known as popular destinations of U.S medical travelers. They decided on medical treatment abroad because other countries can offer cheaper healthcare than in the United States, and several treatment procedures can save up about 30% to 65% in comparison with the similar ones in the U.S. This endowment is difficult to find in the U.S. healthcare system, which is considered to have the most expensive medical costs in the world [4]. In comparison, only 2.2% of Chinese travel abroad (about 3.5 million people) for medical treatment purposes. They often choose Japan, Thailand, South Korea, Taiwan, and India for treatment [5]. The most favored medical procedures sought by medical tourists includes: cosmetic surgery, dentistry, cardiovascular, orthopedics, cancer, reproductive surgeries, weight loss, cans, tests, and health screenings. The motivation of Chinese medical tourists is to seek advanced technology in medical treatment, which is of a better quality in countries that benefit from Western medicine and have a well-regulated healthcare system [6]. According to that, medical tourism is evaluated as a multi-billion dollars industry recently and it continues to grow. The medical tourists around the world are seeking suitable tourism destinations for medical treatment abroad which offer high quality, low cost, specialized care with concierge and hospitality benefits [7]. In fact, the global medical tourism market was valued at USD 16,761 million in 2018, and has been expected to grow to around USD 27,247.6 million by 2024, according to a report of Mordor Intelligence [8]. The report issued by VISA and Oxford Economics also denoted that there are around 11 million medical tourists annually creating a worth of USD 100 billion market values and this offers a huge market with a growth rate at 25% per year [9]. In addition, along with the improvement of living conditions and personal income, the demand of private healthcare consumption, especially in cosmetic surgeries has significantly increased. The data of 2019 addressed that the United States conducted 4.36 million cosmetic procedures, while Brazil had the second largest number with around 2.3 million procedures, followed by Mexico, Germany, India, Italy, Argentina, Colombia, Australia and Thailand [10].

As mentioned above, the business of healthcare travel and medical treatment is booming with the rapid change of business environment and customer demands, the medical services suppliers have to transform and apply new business models in order to meet the demand of foreign patients. 

Today, health services are advertised in the global marketplace with more available “sun and surgery” packages provided by international hospitals or clinic around the world [11]. The competition on global medical tourism industry has been coping with more challenges. In this way, the boost for developing healthcare traveling as a crucial industry is encouraged by governments in some countries, especially in the Asia-Pacific region such as Thailand, South Korea, India, and Taiwan. The developing medical tourism industry has been creating opportunities to improve the local healthcare system and associated economic contributions. This can be achieved by way of reaping more benefits from offering foreign tourists related services, considering the prospect of a “brain drain”, as well as boosting economic growth. In particular, globalization in medical tourism will play an essential role in deciding international patient’s choices, encouraging fair competition among hospitals, and enabling medical tourists high-quality healthcare products [11]. As a result, tourism market players and healthcare services providers, as well as policy makers have been paying much more attention to the business of medical tourism. Therefore, more promotion campaigns on medical services exports have been conducted in this region in the last two decades. Top global medical tourism destinations are recognized as Costa Rica, India, Malaysia, Mexico, Singapore, South Korea, Taiwan, Thailand, Turkey and the United States, as reported by Patients Beyond Borders [12]. Among these, some destinations in the Asia-Pacific region recently dominated the medical tourism market and are expected to continue their stronghold for a few more years. The significant share of the Asia-Pacific region comes from India, Thailand, Singapore, and Malaysia due to the availability of quality healthcare facilities at a comparatively lower cost [13,14]. 

**Figure 1:**
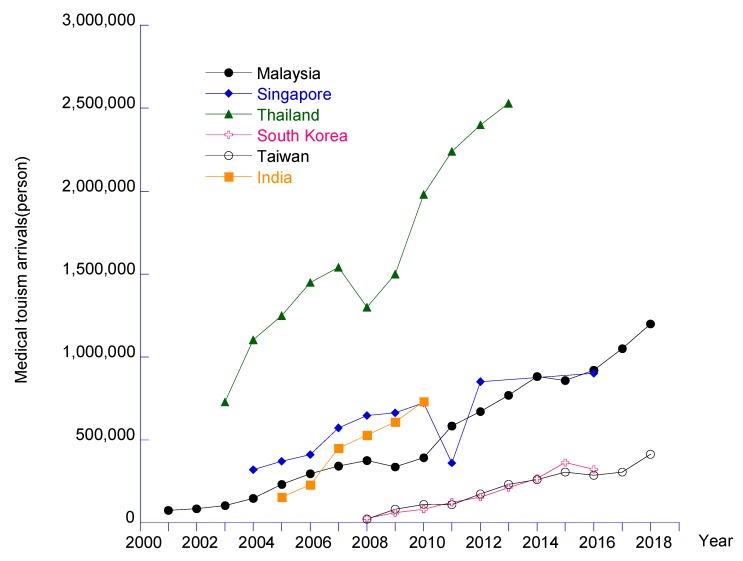
Trends from the medical tourism industry in the Asia-Pacific region. ^1^ Data collected from the following sources: [19,21,22].

The statistical data from Patients Beyond Borders also indicated that Thailand is the most preferred destination which served approximately 2.4 million patients [15], while, India, Malaysia, Taiwan, South Korea were approximately 495,056, 1,050,000, 147,000 and 321,574 patients respectively in 2017 [16,17,18,19,20]. Given the results of increasing international medical tourists toward the Asia-Pacific region, the market revenue of this region was valued at USD 4.8 billion in 2018 [5]. Furthermore, along with the success to make a remarkable accreditation and reputation on global medical services of these providers, the Asia-Pacific region has thus become a major player in the global medical tourism industry with more highly attractive destinations. Figure 1 shows the trend of medical tourism arrivals in the Asia-Pacific region in the period from 2001 to 2018.

As one of favored medical tourism destinations in Asia Pacific region, Taiwan has one of the best healthcare systems in Asia and was ranked as 20th in 2016 MTI Overall ranking with a score of 66.28 [9]. Moreover, this nation was also evaluated a top 10 medical tourism destinations around the world as listed by Healthy Travel Media, the publisher of Patients Beyond Borders.

**Figure 2:**
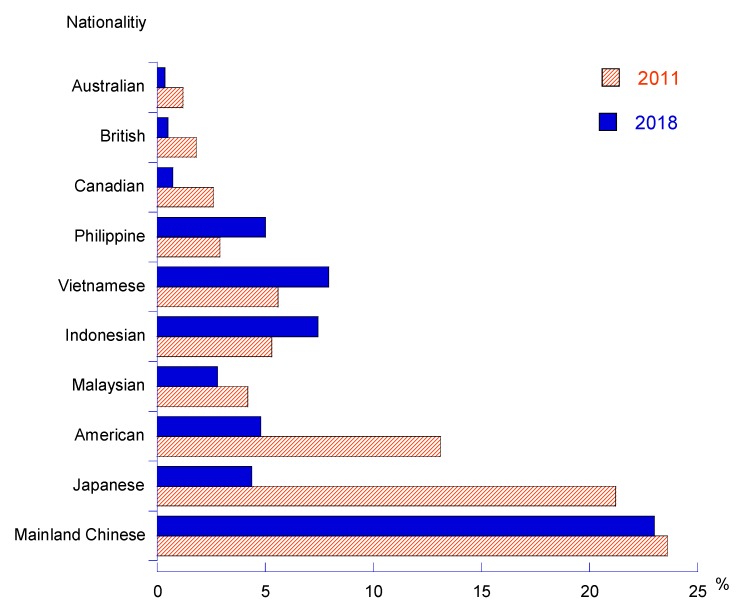
Major medical traveler sources in Taiwan.

**Figure 3:**
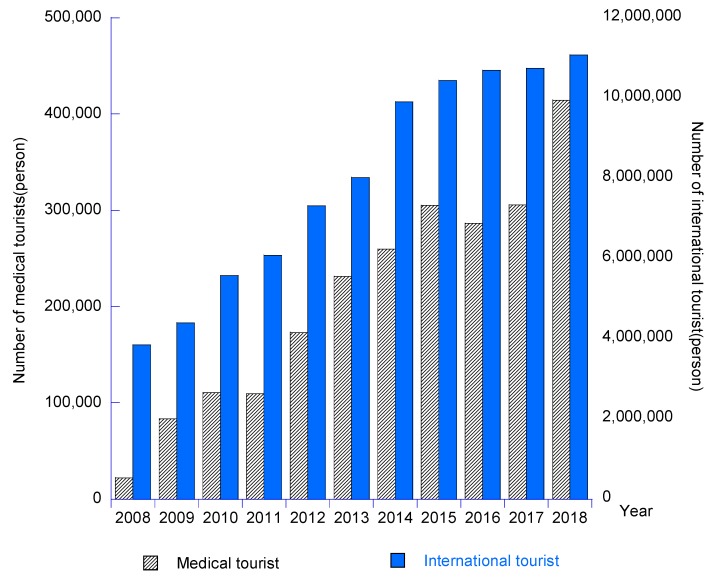
Status of medical tourist and international tourist in Taiwan over the past ten years.

**Figure 4:**
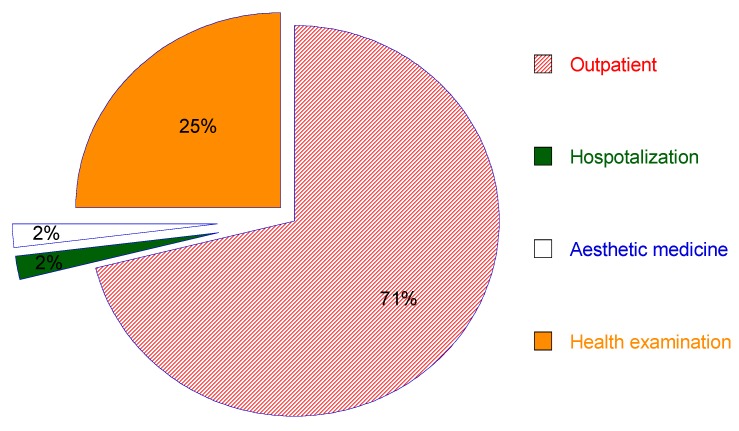
Major healthcare services of international patients in Taiwan.

Although medical and healthcare travel services have created a huge market with profitable contributions directly to Taiwan’s healthcare providers, travel service suppliers and other related industries, Taiwan’s providers are facing more pressures from intense competition and the challenges among international healthcare providers as well as regional rivals. Figure 2, Figure 3 and Figure 4 revealed that the Chinese mainland was the major traveler source of Taiwan’s medical tourism industry. There is a small number of international tourists who come to Taiwan to receive medical and healthcare services. However, there was a big change on medical tourism sources in Taiwan in the past ten years, with Indonesia, the Philippines and Vietnam increasing significantly in recent years, while medical travelers from Japan, the U.S., Canada, Britain and Australasia decreased remarkably. Most of the medical tourists destined for Taiwan intended to receive outpatient and health examination services.

Along with the rapid development of information technologies and the Internet, medical tourism development- a trend of global medical services, and the demand for seeking low-cost healthcare services is increasing [23]. It is also recognized as a major business type of global healthcare services in the future [24]. There is more attention to the sustainable developments of this industry in recent academic research and the media. As aforementioned in the literature, four major themes of patients’ experience emphasized are motivation, decision-making, risks and first-hand accounts. The author also revealed that the high cost of medical services or out-of-pocket payments for procedures in patients’ home countries is a key factor for impacting patients’ decision-making. Additionally, a lack of insurance and long wait-times may also push people into medical tourism. In regards to pull factors, quality is identified as a decisive pull factor, followed by similar languages, sustainability of the political climate, the vacation aspect and international marketing. Furthermore, three types of motivation of medical tourism were recognized as procedure-based, cost-based and travel-based [2]. Some discussions on current issues of medical tourism industry such as the global markets of medical tourists, patients’ choices, providers’ revenue, treatment quality and safety, legal problems, and so on, were reported in [25]. Furthermore, determining the influence of promotional materials and marketing strategies on attracting foreign patients in India or analyzing how Canadian medical travel marketing companies promote healthcare services by websites is also considered in the literature [26,27]. Most recently, issues related to medical tourism marketing in Asian countries were addressed to identify the factors foreign medical tourists on how to choose host countries. Reviewed results presented that perceived service quality and satisfaction play an important role in promoting the development of the medical tourism industry [28]. Besides, human and technological factors were identified as important conditions to develop the medical tourism industry in Malaysia and in developing countries [29].

To analyze the growth and development of this industry with different viewpoints, qualitative techniques were utilized in most of the research [30,31,32,33] based on survey information. For example, a study of [34] used analytic network process (ANP) to learn about the impact of several factors to promote the medical tourism industry. Considered factors included: social factors, medical cost, quality of medical services, and so on. Many factors were engaged, mainly social, economic, and medical ones. Porter’s diamond analysis was adopted for the growth of this industry [24]. DEMATEL-Fuzzy TOPSIS approach was utilized to explore the factors impacting on medical tourism development in Malaysia. Investigated research was conducted with human, technology, and environmental factors based on the data collected from hotel managers [29]. 

In addition, for the market size estimation of the medical tourist industry in general or in Taiwan in particular, there is a small number of studies addressed to propose an effective forecasting model for the medical tourism industry. A recent study of [21] applied three forecasting models to predict the healthcare travelling industry, including the Lotka–Vottera model, the exponential smoothing model, and the Grey model—GM (1,1). While, literature in [35] applied time series models to predict the foreign patients’ arrivals in India up to 2015 based on the data of patient origin. Yet, the findings of these studies cannot be generalized to demonstrate tendency and market volume of this industry presently. 

To sum up, there are a large number of studies focused on determining key factors impacting on the development of medical tourism industry in different host counties or regions. However, to identify the impact factors and evaluate the performance of a destination based on the comparing competitive advantages with the rivals by a statistical approach has not been presented in previous literature yet. In today’s quickly changing international environment, the feedback of market information and customer responses for the first time play a vital role in adjusting the business model of the provider. Thus, it is necessary to have a statistical approach to help policy-makers have an overall viewpoint for market status such as quickly recognizing the determinants of medical tourism industry, competitive advantages of their rivals, as well as to clarify the market changes in the future in the Asia-Pacific region generally. 

Taking the above issues into consideration, this study aims to propose an approach which effectively assesses performance of the medical tourism industry based on a consideration of the economic impact factors. According to that, factors that impact on the sustainable development of medical tourism in the Asia-Pacific region will be identified and the performance of each destination in this region will be also revealed. Some discussion on the medical tourism industry in Taiwan will be considered based on the results of market size predication. The results expect to present an overall perspective of the medical tourism industry in the scope of the Asia-Pacific region and in Taiwan as a destination, particularly. 

**Figure 5:**
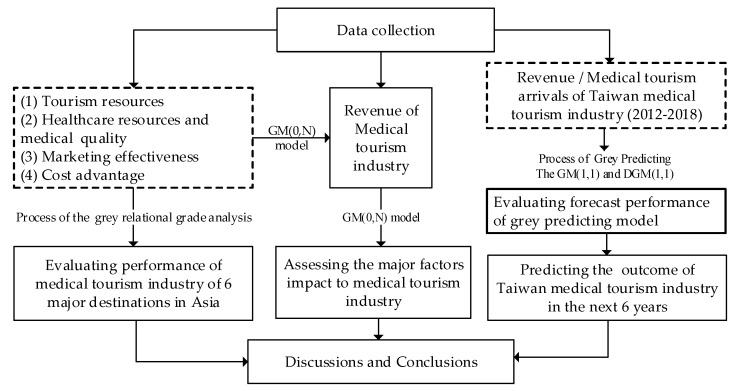
Conceptual framework.

In this study, several mathematical techniques of Grey system theory have been applied as the major methodology. Firstly, the GM (0,1) model is utilized for finding the determinants impact on the revenue of medical tourism industry in the Asia-Pacific region. Secondly, the performance of Thailand, Singapore, Malaysia, India, South Korea and Taiwan is analyzed and ranked using Grey relational analysis (GRA). Thirdly, the trend and market size of the medical tourism industry of Taiwan in the next six years will be shown by applying the Grey predicting analyses. Finally, some discussions and future directions of business strategies for sustainable development of Taiwan medical tourism industry will be suggested based on research results. The conceptual framework of this study is presented in Figure 5. 

Followed by this section, the mathematical functions of the analytical approach are presented in the next section. Results and discussions are shown in Section 3 and Section 4. Conclusions are presented in the last section.

## 2. Methodology

### 2.1. Literatures Review on Grey System Theory

The Grey system theory was first established in 1989 by Deng. This system offers the techniques, notions and ideas to analyze latent and intricate systems [36]. With the strengths on non- functional series for model construction and simple calculation, Grey system theory has been utilized for factor analyzing, optimization theorem, forecasting or numerical analyzing in different research fields [37,38,39,40]. Grey generating, Grey relational analysis, Grey model construction, Grey forecasting, Grey decision making and Grey control are major sections of Grey system theory, which was widely used for academic studies and practical industries such as engineering [41,42], tourism [43], and service industries [44], etc. 

Grey relational analysis (GRA) is commonly referred to as a method for measuring the relationship among different discrete sequences. This technique can be effective in accessing information from different database. Nevertheless, there are different characteristics of data (quantitative or qualitative). Thus, effective applications can reduce manpower cost, and improve efficiency and work quality [41]. In fact, based on the application of GRA on selecting the selecting vendors, a study [44] indicated that GRA obviously reduced purchasing cost and increased production efficiency and overall competitiveness. Owing to these advantages, the application of GRA has been widely adopted. For example, a study [45] utilized the GRA to explore the relationship between different sources of air pollutions and transportation activities in Japan. The research of [46] was applied with GRA to determine passengers’ shopping preferences and satisfaction with airport retailing products. Still now, applications and improvements and expansions of GRA have been continuously proposed [47].

Based on Grey model construction, the GM (0,N) is known as the Grey model with n variables and the one first-order equation or GM (1,N) models is defined as a Grey model with n variables and a zero-order equation. These models are often used for multivariate correlation analysis in a Grey system. These models have been applied popularly in current studies [46]. Literature in [48] adopted GM (0,N) and GM (1,N) to determine the relationship between different skin physiological factors impact on skin characteristics based on a weighting of these factors.

In addition, one of the crucial parts of Grey system theory is Grey forecasting. Grey forecasting is a high accuracy, short-term forecasting technique, which is successful in its application to various fields [38,49]. It can be employed in situations with limited data—down to as little as four observations [21]. For this reason, Grey forecasting can be used as an effective tool for understanding the uncertain environment under the limitation of information [50]. 

As per the discussions above, and according to the conceptual framework in the previous section, some techniques of the Grey system include the process of the GM (0,N) model, GRA, and the Grey forecasting GM (1,1) model and the discrete Grey model—DGM (1,1) model which are applied to find the main factors impacting on the medical tourism industry in the Asia-Pacific region, industry performance of each medical tourism destination, future change of medical tourists and outcome of Taiwan’s medical tourism respectively. Thus, in the next section, we present the mathematical functions and procedures of GRA, the GM (0,N) model, the GM (1,1) model and the DGM (1,1) model. Detail demonstrations are shown below. 

### 2.2. The GM (0,N) Model

In Grey system theory, the model of GM (0, N) is described as a special case of GM (1, N). Normally, this model is utilized to determine the quantitative relations among the N variables, which is adopted for static factor analysis. The mathematical function of GM (0,N) is presented as follows.

(1) $$az^{\left(1\right)}\left(k\right)=\overset{n}{\underset{i=2}{\sum }}b_{j}x_{j}^{\left(1\right)}\left(k\right)=b_{2}x_{2}^{\left(1\right)}\left(k\right)+b_{3}x_{3}^{\left(1\right)}\left(k\right)+\ldots +b_{n}x_{n}^{\left(1\right)}\left(k\right)$$

where, $z_{1}^{\left(1\right)}(k)=0.5x_{1}^{\left(1\right)}(k-1)+0.5x_{1}^{\left(1\right)}(k)$, k=2,3…n

Step 1: Substituting each coefficient into Equation (1) to obtain:

(2) $$\begin{array}{l} az_{1}^{\left(1\right)}(1)=b_{2}x_{2}^{\left(1\right)}(2)+\ldots +b_{n}x_{n}^{\left(1\right)}(2)\\ az_{1}^{\left(1\right)}(3)=b_{2}x_{2}^{\left(1\right)}(3)+\ldots +b_{n}x_{n}^{\left(1\right)}(3)\\ \ldots \\ az_{1}^{\left(1\right)}(n)=b_{2}x_{2}^{\left(1\right)}(n)+\ldots +b_{n}x_{n}^{\left(1\right)}(n) \end{array}$$

Step 2: Dividing elements in Equation (2) by a1 and translate them into a matrix form:

(3) $$\begin{array}{l} \left[\begin{array}{l} 0.5x_{1}^{\left(1\right)}(1)+0.5x_{1}^{\left(1\right)}(2)\\ 0.5x_{1}^{\left(1\right)}(2)+0.5x_{1}^{\left(1\right)}(3)\\ \vdots \\ 0.5x_{1}^{\left(1\right)}(n-1)+0.5x_{1}^{\left(1\right)}(n) \end{array}\right]=\left[\begin{array}{ccc} x_{2}^{\left(1\right)}(2) & \ldots & x_{n}^{\left(1\right)}(2)\\ x_{2}^{\left(1\right)}(3) & \ldots & x_{2}^{\left(1\right)}(3)\\ \vdots & \ldots & \vdots \\ x_{2}^{\left(1\right)}(n) & \ldots & x_{n}^{\left(1\right)}(n) \end{array}\right]\left[\begin{array}{c} \frac{b_{2}}{a_{1}}\\ \frac{b_{3}}{a_{1}}\\ \frac{b_{4}}{a_{1}}\\ \vdots \\ \frac{b_{n}}{a_{1}} \end{array}\right] \end{array}$$

Let $\frac{b_{j}}{a_{1}}={\hat{b}}_{m}$, where, m=2,3,…n, and then Equation (3) can be rearranged as following:

(4) $$\begin{array}{l} \left[\begin{array}{l} 0.5x_{1}^{\left(1\right)}(1)+0.5x_{1}^{\left(1\right)}(2)\\ 0.5x_{1}^{\left(1\right)}(2)+0.5x_{1}^{\left(1\right)}(3)\\ \vdots \\ 0.5x_{1}^{\left(1\right)}(n-1)+0.5x_{1}^{\left(1\right)}(n) \end{array}\right]=\left[\begin{array}{ccc} x_{2}^{\left(1\right)}(2) & \ldots & x_{n}^{\left(1\right)}(2)\\ x_{2}^{\left(1\right)}(3) & \ldots & x_{2}^{\left(1\right)}(3)\\ \vdots & \ldots & \vdots \\ x_{2}^{\left(1\right)}(n) & \ldots & x_{n}^{\left(1\right)}(n) \end{array}\right]\left[\begin{array}{c} {\hat{b}}_{2}\\ {\hat{b}}_{3}\\ {\hat{b}}_{4}\\ \vdots \\ {\hat{b}}_{n} \end{array}\right] \end{array}$$

Step 3: The values of ${\hat{b}}_{m}$ can be calculated by way of inverse matrix, shows as below:

(5) $$\hat{B}={\left(P^{T}P\right)}^{-1}P^{T}Q$$

where,

$$P=\left[\begin{array}{ccc} x_{2}^{\left(1\right)}(2) & \ldots & x_{n}^{\left(1\right)}(2)\\ x_{2}^{\left(1\right)}(3) & \ldots & x_{2}^{\left(1\right)}(3)\\ \vdots & \ldots & \vdots \\ x_{2}^{\left(1\right)}(n) & \ldots & x_{n}^{\left(1\right)}(n) \end{array}\right];Q=\left[\begin{array}{l} 0.5x_{1}^{\left(1\right)}(1)+0.5x_{1}^{\left(1\right)}(2)\\ 0.5x_{1}^{\left(1\right)}(2)+0.5x_{1}^{\left(1\right)}(3)\\ \vdots \\ 0.5x_{1}^{\left(1\right)}(n-1)+0.5x_{1}^{\left(1\right)}(n) \end{array}\right];\hat{B}=\left[\begin{array}{c} {\hat{b}}_{2}\\ {\hat{b}}_{3}\\ {\hat{b}}_{4}\\ \vdots \\ {\hat{b}}_{n} \end{array}\right]$$

Accordingly, the association among variables can be established and the weighting and ranking of factors can also be determined by judging against the value of ${\hat{b}}_{m}$.

### 2.3. Grey Relational Analysis

#### 2.3.1. Traditional Grey Relational Grade

The formula of Grey relational grade which was proposed by Deng (1988) as following:

Step 1: Compute the Grey relational grade. Suppose a target sequence with k entities:

(6) $$x_{0}=\left\{x_{0}(1),x_{0}(2),x_{0}(3),\ldots x_{0}(k)\ldots x_{0}(n)\right\}$$

where, $x_{i}$ is the inspected sequences,

(7) $$x_{i}=\left\{x_{i}(1),x_{i}(2),x_{i}(3),\ldots x_{i}(k)\ldots x_{i}(n)\right\},\;i=1,2,\ldots ,m$$

where, $x_{i}$ must be satisfied by three conditions, that consist of (a) non-dimension; (b) scaling and (c) polarization.

Further then, the Grey relational grade is determined as follows:

(8) $$\gamma (x_{i}(k),x_{j}(k))=\frac{\Delta_{\min.}+\varsigma \Delta_{\max.}}{\Delta_{0i.}(k)+\varsigma \Delta_{\max.}}$$

where,

i=1,2,…,m, k=1,2,…,n, j∈I
$\Delta_{0i}(k)=\left|x_{0}(k)-x_{i}(k)\right|$
$\Delta_{\min.}=\min_{i}\min_{k}\Delta_{0i}(k)$
$\Delta_{\max.}=\max_{i}\max_{k}\Delta_{0i}(k)$
ς is the distinguishing coefficient, ς=[0,1], normally this value is taken as 0.5.

Taking the mean of the Grey relational coefficient then, the Grey relational grade $\varpi_{0i}$ for sequences $x_{0}$ to $x_{i}$ is calculated as follows:

(9) $$\omega_{0i}=\frac{1}{n}\sum_{k=1}^{n}\gamma (x_{i}(k),x_{j}(k))$$

Notice that when the range of the sequence is too large or the standard value is too enormous, the Grey relational generation process can be considered to treat the data sequences before calculating the Grey relational grade. 

Let $x_{i}^{*}(k)$ be a generated value of the Grey relational generation. Three situations of Grey relational generation which is popularly used for this process is shown as follows [51]:

Upper bound effectiveness measurement:

(10) $$x_{i}^{*}(k)=\frac{x_{i}^{\left(0\right)}(k)-\min_{k}x_{k}^{\left(0\right)}}{\max_{k}x_{i}^{\left(0\right)}(k)-\min_{k}x_{k}^{\left(0\right)}}$$

Lower bound effectiveness measurement:

(11) $$x_{i}^{*}(k)=\frac{\max_{k}x_{i}^{\left(0\right)}(k)-x_{i}^{\left(0\right)}(k)}{\max_{k}x_{i}^{\left(0\right)}(k)-\min_{k}x_{k}^{\left(0\right)}}$$

Moderate effectiveness measurement:

(12) $$x_{i}^{*}(k)=\frac{\left|x_{i}^{\left(0\right)}(k)-x_{0}(k)\right|}{\max\left\{\max_{k}x_{i}^{\left(0\right)}(k)-x_{0}(k)-\min_{k}x_{k}^{\left(0\right)}\right\}}$$

where $\min_{k}x_{k}^{\left(0\right)}$ and $\max_{k}x_{k}^{\left(0\right)}$ are the minimum and maximum values of $x_{i}(k)$ respectively.

Step 2: Ordering the Grey relational rank:

According to the value of ϖ, we can order the rank of Grey relational grade. If $\varpi_{0i}\geq \varpi_{0j}$, the Grey relational rank of $x_{i}$ is greater than Grey relational rank of $x_{j}$. 

According to the purpose of this study and in line with the mention as the previous section, the mathematical function of the Grey model GM (1,1) and the DGM (1,1) model are presented in this section.

#### 2.3.2. The GM (1,1) Model

The GM (1,1) model is understood as Grey model first order, one variable. The mathematical function of GM (1,1) is described as below:

Step 1: Suppose an original sequence with n entries is $x^{\left(0\right)}$

(13) $$x^{\left(0\right)}=\left\{x^{\left(0\right)}(1),x^{\left(0\right)}(2),x^{\left(0\right)}(3),\ldots ,x^{\left(0\right)}(i),\ldots x^{\left(0\right)}(n)\right\}$$

where, $x^{\left(0\right)}(i)$ is the value at time i (i=1,2,…n)

Step 2: Generating original sequences by using one-time accumulated generating operation (1-AGO), the generated sequences $x^{\left(1\right)}$ are:

(14) $$x^{\left(1\right)}=\left\{x^{\left(1\right)}(1),x^{\left(1\right)}(2),\ldots ,x^{\left(1\right)}(i),\ldots ,x^{\left(1\right)}(n)\right\}$$

where, $x_{}^{\left(1\right)}(i)=\sum_{j=1}^{i}x_{}^{\left(0\right)}(j)$

Step 3: A first-order differential equation with one variable is obtained as following:

(15) $$\frac{dx_{}^{\left(1\right)}}{dt}+ux_{}^{\left(1\right)}=v$$

where, u, v are the developing coefficient and the Grey input coefficient, respectively. These two coefficients can be determined by the least squares method as in the following:

(16) $${\left[u,v\right]}^{T}={\left(P^{T}P\right)}^{-1}P^{T}H$$

where,

$$P=\left[\begin{array}{c} -\left(x^{\left(1\right)}(1)+x^{\left(1\right)}(2)\right)/2\\ -\left(x^{\left(1\right)}(2)+x^{\left(1\right)}(3)\right)/2\\ \cdots \\ -\left(x^{\left(1\right)}(n-1)+x^{\left(1\right)}(n)\right)/2 \end{array}\;\begin{array}{c} 1\\ 1\\ \cdots \\ 1 \end{array}\right]$$

$$H={\left[x^{\left(0\right)}(2),x^{\left(0\right)}(3),\ldots ,x^{\left(0\right)}(n)\right]}^{T}$$

Therefore, the solution of Equation (15) is estimated as:

(17) $$x^{\left(1\right)}\left(i\right)=\left[x^{\left(1\right)}\left(1\right)-\frac{v}{u}\right]e^{-ut}+\frac{v}{u}$$

Equation (16) is also known as time response function of the differential equation. From Equation (17), the time response function of the GM (1,1) is expressed by:

(18) $${\hat{x}}^{\left(1\right)}\left(i\right)=\left[x^{\left(0\right)}\left(1\right)-\frac{v}{u}\right]e^{-u\left(k-1\right)}+\frac{v}{u}\;\left(i=1,2,\ldots n\right)$$

Further, the predicted value ${\hat{x}}^{\left(0\right)}(i)$ can be obtained by utilizing the operation of one- time inverse accumulated generating operation (1-IAGO), as in the following:

(19) $${\hat{x}}^{\left(0\right)}\left(i\right)={\hat{x}}^{\left(1\right)}\left(i\right)-{\hat{x}}^{\left(1\right)}\left(i-1\right)\;\left(i=2,3,\ldots ,n\right)$$

where, ${\hat{x}}^{\left(0\right)}(1)={\hat{x}}^{\left(1\right)}(1)$

#### 2.3.3. DGM (1,1) Model

The discrete Grey model, namely the DGM (1,1) model, is referred to as the Grey model with one variable and one first-order equation. The mathematical function of DGM (1,1) is described as below: 

Suppose an original sequence with n entries is:

(20) $$x^{\left(0\right)}=\left\{x^{\left(0\right)}(1),x^{\left(0\right)}(2),x^{\left(0\right)}(3),\ldots ,x^{\left(0\right)}(t),\ldots x^{\left(0\right)}(n)\right\}$$

where, $x^{\left(0\right)}(t)$ is the value at time t (t=1,2,…n) Generating original sequences by using one-time accumulated generating operation (1-AGO), the generated sequences $x^{\left(1\right)}$ are:

(21) $$x^{\left(1\right)}=\left\{x^{\left(1\right)}(1),x^{\left(1\right)}(2),\ldots ,x^{\left(1\right)}(t),\ldots ,x^{\left(1\right)}(n)\right\}$$

where, $x_{}^{\left(1\right)}(t)=\sum_{j=1}^{t}x_{}^{\left(0\right)}(j)$

Step 3: A first-order differential equation with one variable is obtained as following:

(22) $$\frac{dx_{}^{\left(1\right)}}{dt}+\omega x_{}^{\left(1\right)}=\gamma$$

where, ϖ, γ are the developing coefficient and the Grey input coefficient, respectively. These two coefficients can be determined by the least squares method as in the following:

(23) $${\left[\omega ,\gamma \right]}^{T}={\left(M^{T}M\right)}^{-1}M^{T}N$$

where,

$$M=\left[\begin{array}{c} -\left(x^{\left(1\right)}(1)+x^{\left(1\right)}(2)\right)/2\\ -\left(x^{\left(1\right)}(2)+x^{\left(1\right)}(3)\right)/2\\ \cdots \\ -\left(x^{\left(1\right)}(n-1)+x^{\left(1\right)}(n)\right)/2 \end{array}\;\begin{array}{c} 1\\ 1\\ \cdots \\ 1 \end{array}\right]$$

$$N={\left[x^{\left(0\right)}(2),x^{\left(0\right)}(3),\ldots ,x^{\left(0\right)}(n)\right]}^{T}$$

Therefore, the solution of Equation (23) is estimated as:

(24) $${\hat{x}}^{\left(1\right)}\left(t+1\right)=\omega^{t}x^{\left(0\right)}(1)+\frac{1-\omega^{t}}{1-\omega }\gamma$$

where ${\hat{x}}^{\left(1\right)}(t)$ is generated sequences by 1-AGO processing.

The restored values of ${\hat{x}}^{\left(0\right)}(t)$ can be given by:

(25) $${\hat{x}}^{\left(0\right)}\left(t+1\right)={\hat{x}}^{\left(1\right)}\left(t+1\right)-x^{\left(1\right)}(t)=\left(x^{\left(0\right)}(1)-\frac{\gamma }{1-\omega }\right)\left(1-\frac{1}{\omega }\right)\omega^{t}$$

where, ${\hat{x}}^{\left(0\right)}(1)={\hat{x}}^{\left(1\right)}(1)$

#### 2.3.4. Evaluation of the Capability of the Proposed Model

For assessing the performance of proposed prediction models, three statistical measures are applied for comparing the accuracy of each model based on the actual value $x^{\left(0\right)}(i)$ and the predicted value ${\hat{x}}^{\left(0\right)}(i)$ and $\hat{x}\prime^{\left(0\right)}(i)$. These are relative percentage error (RPE), the mean absolute percentage error (MAPE) and the prediction accuracy (ρ), respectively. These indexes are defined as below:

(26) $$RPE=\frac{\left|{\hat{x}}^{\left(0\right)}(i)-{{\hat{x}}^{\prime }}^{\left(0\right)}(i)\right|}{{\hat{x}}^{\left(0\right)}(i)}\times 100\%$$

(27) $$MAPE=\left(\frac{1}{n}\sum_{i=1}^{n}\frac{\left|{\hat{x}}^{\left(0\right)}(i)-{{\hat{x}}^{\prime }}^{\left(0\right)}(i)\right|}{{\hat{x}}^{\left(0\right)}(i)}\right)\times 100\%$$

(28) ρ=1−MAPE

The capability of forecast will be evaluated by four grades of forecast performance, including unqualified (≤85%), qualified (>85%), good (>90%) and excellent (>95%) evaluations [52].

### 2.4. Data Preparation

In link with the main purposes of this study, the collected data was divided into three parts.

For the first part, we used the GM (0,1) model for finding the major factors impacting on medical tourism arrivals. Based on discussions in literatures [25,26,34], we collected several economic indicators related to sustainable development for medical tourism including tourism resources, healthcare resources and medical quality, marketing effectiveness and medical cost advantage. Measurements of tourism resources consist of the number of the international tourist arrivals, the number of low-cost airlines companies, the travel and tourism competitiveness index. Four measurements of for healthcare resources and medical quality consist of the number of physicians (per 1000 people), current health expenditure per capita in $USD, number of hospital beds per 1000 people, and number of hospitals with joint commission of international accreditation. For marketing effectiveness and medical cost advantage, we used the number of medical tourism agencies and facilitations, and average prices of some common medical procedure to measure. An amount of nine variables were selected as independent variables, while, the revenue of medical tourisms of six destinations was adopted as the dependent variable. The data is presented in Table 1.

For the second part, the GRA was employed to evaluate the industry performance of six destinations of medical tourism in the Asia-Pacific region, including Singapore, Thailand, Malaysia, South Korea and India. The variables from the first to the ninth of Table 1 data will be analyzed by GRA processing. 

For the third part, the GM (1,1) model and DGM (1,1) model was applied to estimate the market size of Taiwan’s medical industry in the next six years. The data, including medial tourism revenue in the period from 2012 to 2018 were collected to construct forecasting models. 

## 3. Results

**Figure 6:**
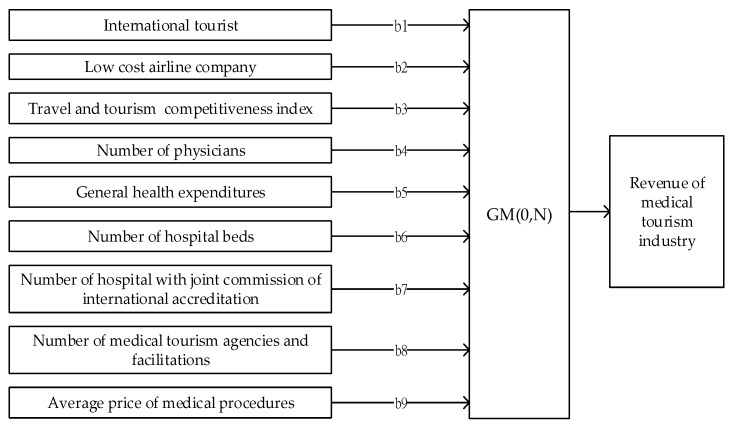
Analyzing framework of the Grey model (GM, 0,N) model.

This section presents the results of GM (0,N), GRA and Grey forecasting GM (1,1) and DGM (1,1) models, respectively. In order to find the impact factors of medical tourism industry, the process of GM (0,N) was used. The analysis framework is described in Figure 6.

Based on the obtained data in Table 1, let $x_{1}^{\left(0\right)},x_{2}^{\left(0\right)},x_{3}^{\left(0\right)},x_{4}^{\left(0\right)},x_{5}^{\left(0\right)},x_{6}^{\left(0\right)},x_{7}^{\left(0\right)},x_{8}^{\left(0\right)},x_{9}^{\left(0\right)},x_{10}^{\left(0\right)}$ represent the variables of number of the international tourist arrivals, the number of low-cost airlines companies, the travel and tourism competitiveness index, the number of physicians, current health expenditure per capita, number of hospital beds, number of hospitals with joint commission of international accreditation, the number of medical tourism agencies and facilitations, average prices of some common medical procedure, and revenue of medical tourism industry, respectively. Thus, the original series of collected factors was built as follows:

$x_{1}^{\left(0\right)}=(35592,13903,\;25948,\;13336,\;15543,\;10740)$
$x_{2}^{\left(0\right)}=(6,2,2,6,5,1)$
$x_{\left(3\right)}^{\left(0\right)}=(4.5,4.8,4.5,4.8,4.4,4.3)$
$x_{4}^{\left(0\right)}=(0.8,2.3,1.5,3.7,0.8,2.2)$
$x_{5}^{\left(0\right)}=(221.92,2462.39,361.52,2043.86,62.72,2732)$
$x_{6}^{\left(0\right)}=(2.1,2.4,1.9,11.5,0.8,5.7)$
$x_{7}^{\left(0\right)}=(68,20,13,24,39,13)$
$x_{8}^{\left(0\right)}=(8,2,5,2,112,1)$
$x_{9}^{\left(0\right)}=(11808,12690,8025,14883,5783,10167)$
$x_{10}^{\left(0\right)}=(600,150,350,655,450,300)$

Applying the process of the GM (0,N) model, the results of factor analyzing are presented in Table 2. 

Accordingly, tourism resources and healthcare resources-medical quality play the crucial role for boosting the sustainable development of the medical tourism industry. While, the marketing effectiveness and medical cost have been identified as auxiliary effects. In particular, the travel and tourism competitiveness, the number of physicians, the number of hospital beds and the number of low-cost airlines companies strongly impact the revenue of this industry.

Three elements of tourism resources consist of international arrivals, airline companies and travel and tourism competitiveness impacted to medical tourist arrivals in which the element weightings are 0.1823, 0.1823 and 1515.4284, respectively. Responding to element ranking is placed at eighth, fourth and first respectively. In addition, four elements of healthcare resources and medical quality significantly present as major conditions for developing international healthcare services. The element weightings of the number of physicians, current health expenditure per capita, the number of hospital beds, and the number of hospitals with the joint commission of international accreditation are 1444.9664, 0.1833, 191.8731, and 0.7906, respectively. Responding to element ranking is the ranking at the second, seventh, third and sixth places. While, marketing effectiveness and medical cost placed at fifth and ninth positions, respectively, among nine considered elements. The factor weights are 1.0052 and 0.0585 respectively.

In the second part, the data showed in Table 2 was adopted to assess the performance of six destinations of medical tourism in the Asia-Pacific region including Thailand, Singapore, Malaysia, South Korea and India, respectively. Ten collected variables and situation of Grey relational generation for sustainable development on medical tourism were conducted as shown in Table 3. The analyzing was adopted by Grey relational analysis. 

Firstly, before conducting the GRA process, the data is treated by the Grey relational generation process as noted in Section 2.2 and in order to find out the standard sequences namely, X0={35592,6, 4.8, 3.7, 2732, 11.5, 68, 112, 5783,655} Further, by applying the formula of GRA, we calculate the Grey relational grade ω. The results and the responding eigenvalues are presented as $\varpi_{j}=\left\{0.966152,0.893754,0.944011,0.893026,0.915399,0.885301\right\}$. The eigenvalue would be revealed the overall performance of each selected destination.

According to the theory of the Grey relational grade, the ranking of six destinations are arranged based on the its eigenvalues as x1 = 0.966152 > x2 = 0.944011 > x5 = 0.915399 > x2 = 0.893754 > x4 = 0.893026 > x6 = 0.885301. Given this result, the eigenvalues of Thailand, Malaysia, India, Singapore, South Korea and Taiwan are 0.966152, 0.893754, 0.944011, 0.893026, 0.915399 and 0.885301, respectively. The ranking of six destinations based on GRA are shown in Table 4.

Based on the results of GRA, the performance of Thailand achieved the leading position, followed by Malaysia, India, Singapore, South Korea, and Taiwan. 

**Figure 7:**
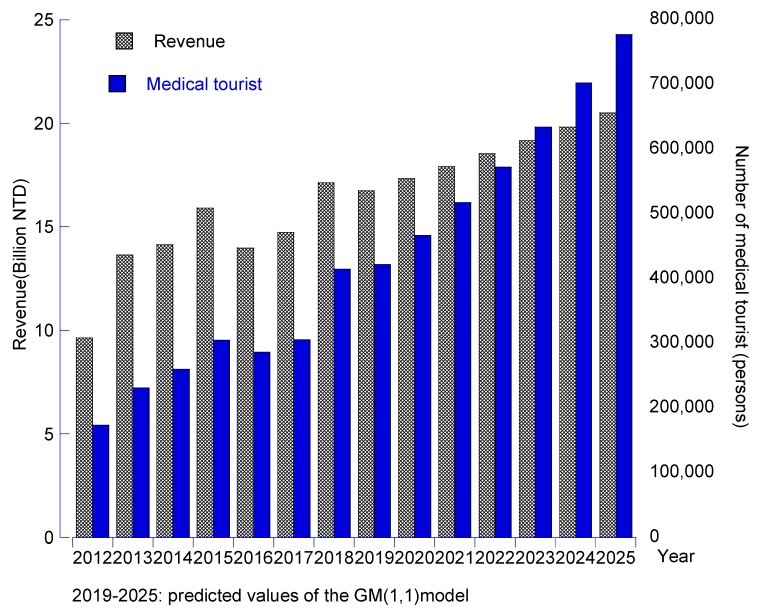
The market size of Taiwan’s medical tourism industry over the next six years.

In the third part, to realize the market size of Taiwan’s medical tourism industry, this study used the data of annual revenues and number of medical travelers in the period from 2012 to 2018, construct forecasting models as a Grey predicting process. Two predicting models were applied, namely the GM (1,1) model and the DGM (1,1) model. Based on these results, the value of some statistical measures including the relative percentage error (RPE), mean average of percentage error (MAPE), model accuracy (p), and predictive capability were calculated to evaluate the performance of proposed forecasting models. The obtained results are summarized in Table 5 and Figure 7.

According to the obtained results from Table 5, the MAPE value of the traditional GM (1,1) model and the DGM (1,1) model was ranked from 4.75% to 6.75%. That implied the accuracy rate or proposed models was, in ranked form; 93.25% and 95.25%. The achieved values of accuracy rate revealed that the two proposed Grey models have high performance in predicting capability according to [52]. For this reason, the future market of the Taiwan medical tourism is also estimated by the two models, the results showed in the Table 6.

Revenue from Taiwan has increased slowly in the last six years, with the market worth approximately NT$20.5 billion, and the number of medical travelers is expected to increase to 777,523 by 2025.

## 4. Discussion

The Asia-Pacific region is known as a common medical tourism destination which is successful at attracting global medical travelers and is placed sixth of the world’s top ten. Taiwan is a one of a favor healthcare destination lying in this region. With the encouragement of Taiwan’s government, cross-border healthcare and medical services have rapidly developed since 2008. Taiwan has a world-class healthcare system and they receive some high difficulty surgeries such as liver transplantation, cord blood transplantation, cancer treatment and plastic surgeries. However, most medical tourists to Taiwan go with the intention to receive outpatient and health examination services as per the data outlined in Figure 2, and the majority of medical travelers come from China Mainland, Indonesia, Philippine and Vietnam as presented in Figure 1. In addition, the forecasting results in Table 6 also indicated that the revenues from the Taiwan medical tourism industry have expected slow increases in the next six years, and the market worth is estimated to be close to NT$20.5 billion dollars by 2025. 

Nevertheless, the results of GRA ensured that, Taiwan’s medical tourism has ranked in sixth place compared with other five regional providers. Results of GM (0,1) also confirmed tourism resources as well as healthcare and medical infrastructures are recognized as vital conditions for sustainable development of international medical services in a host country. However, medical tourism related costs are always the key factor when the patient selects a medical provider, besides treatment quality. Moreover, along with the quick development of the Internet, the role of marketing has become an important channel for promoting medical services toward foreign patients.

The rapid growth of regional rivals with more competitive advantages on low cost, high quality, flexible procedures, effective marketing and less legal restrictions has strongly affected the market share of Taiwan on the global medical market. Furthermore, beside Thailand—Singapore, Malaysia, and South Korea are the major competitive providers for Taiwan in the recent period. The market share for plastic surgeries are expected increase for South Korea due to competitive pricing and marketing effectiveness as discussed in [43]. However, Thailand has placed as the top destination with the largest number of foreign patients in Asia-Pacific as noted above.

Thus, in order to attract more and more medical travelers, as well as retain competitive advantages and sustainable development in the future, Taiwan medical tourism players should be considered as strengthening marketing strategies and cost-competitiveness. Moreover, expanding foreign patient sources to different regions as well as attracting more wealthy patients with luxury healthcare packages can be promoted. On the other side, the Government also plays a crucial role in enhancing the competitiveness of Taiwan’s medical tourism industry via formulating appropriate legislations and integrating with other industries to offer more conveniences as much as reducing the risk for medical tourists such as services information, lower air fares, tax returns, cross-border insurances, and reimbursement, etc.

Although the Asia-Pacific region is known as a favorite destination of medical tourists recently, the sustainable development should be focused on improving healthcare resources and medical quality, tourism environment and its competition as well as marketing effectiveness, besides on cost advantage. Compared to the previous studies [2], despite using different methods to explore the factors impacting on the development of the medical tourism industry, the finding of this study provides the same information for market players in this industry. The motivation of an international tourist to become a medical traveler is to seek low-cost healthcare and medical services abroad, but the quality of medical tourism providers is the first condition which is considered by medical traveler. According to that, improving the healthcare system toward international levels as well as enhancing international images of host countries will also contribute to attract international tourist and medical travelers.

## 5. Conclusions

Results of GM (0, N) confirmed that tourism resources and attribution as well as healthcare and medical infrastructures are vital conditions for sustainable development of international medical services in the Asia-Pacific region. Moreover, performance analysis identified that Thailand has great performance and retained the top destination for sustainable development of healthcare travel, followed by Malaysia, India, Singapore, South Korea and Taiwan.

The Taiwanese healthcare travel industry takes advantage of its best healthcare infrastructures and world-class medical expertise as well as high reputation medical treatments. However, based on assessing the competitive advantages of Taiwan and regional destinations, the results of GRG analysis indicated that Taiwan has a low performance comparing to other regional rivals. Forecasting results have proven that healthcare travel revenue of Taiwan is expected to increase slowly in the next five years, in which the market worth will grow to approximately NT$20.5 billion dollars. The number of medical travelers will be expected to increase to 777,523 in 2025. In the long run, in order to not miss the opportunities for this lucrative market, improving for competitive strategies on treatment cost and a propagating marketing would be expected by healthcare providers with efforts from Taiwan’s government.

This study contributes to propose an approach which effectively assesses performance of the medical tourism industry based on considering the economic impact factors. Grey system theory is utilized as a major analyzing approach. According to that, factors impact on the sustainable development of medical tourism in the Asia- Pacific region were identified and the performance of each destination in this region was also revealed. The results presented an overall perspective of the medical tourism industry in the scope of the Asia-Pacific region and in Taiwan destination in particularly. 

The findings of this study are expected to provide useful information for key players of the medical tourism industry in strategic planning. The conceptual framework and the application of Grey system theory can be implicated in other industries in order to explore market information.

For estimation of the future market size of each destination in Asia-Pacific region, this study only focused on the number of medical travelers and the revenue of Taiwan, due to the data limitation of the rest five destinations. In the future, if data is available, expanded research can be considered with a global market scope as well as being conducted by each destination.

## Figures and Tables

**Table 1:** Collected measurements and data for analyzing the impact factors of medical tourism industry in the Asia-Pacific region and evaluating performance of each destination.

Evaluated factors	Measurements	Variable Rename	Destinations					
			Thailand	Singapore	Malaysia	South Korea	India	Taiwan
Tourism resources	Number of international tourist arrivals (1000 million persons) [53] 2017)	X1	35,592	13,903	25,948	13,336	15,543	10,740
	Number of low-cost airlines companies [54]	X2	6	2	2	6	5	1
	Travel and Tourism Competitiveness Index [55]	X3	4.5	4.8	4.5	4.8	4.4	4.3
Healthcare resource and medical quality	Number of physicians (per 1000 people) (2017) [56]	X4	0.8 (2017)	2.3 (2016)	1.5 (2015)	3.7 (2017)	0.8 (2017)	2.2
	Current Health Expenditure per capita $US [57]	X5	221.92 (2016)	2462.39 (2016)	361.52 (2016)	2043.86 (2016)	62.72 (2016)	2732 (2014)
	Number of hospital beds per 1000 people [56,58]	X6	2.1 (2010)	2.4 (2015)	1.9 (2015)	11.5 (2015)	0.8 (2016)	5.7 (2017)
	Number of hospitals with joint commission of international accreditation [59]	X7	68	20	13	24	39	13
Marketing effectiveness	Number of medical tourism agencies and facilitations [60]	X8	8	2	5	2	112	1
Cost Advantage	Average price of medical procedures (US $) [61]	X9	11,808	12,690	8025	14,883	5783	10,167
Medical tourism industry	Medical tourism revenue (US $ million) [62]	X10	600	150	350	655	450	300

**Table 2:** The results of factor analysis by the GM (0,N) model.

Evaluated Factors	Measurements	Element Weighting	Ranking
Tourism resources	Number of international tourist arrivals	0.1823	8
	Number of low-cost airlines companies	0.1823	4
	Travel and Tourism Competitiveness index	1515.4284	1
Healthcare resources and medical quality	Number of physicians	1444.9664	2
	Current Health Expenditure per capita $US	0.1833	7
	Number of hospital beds	191.8731	3
	Number of hospitals with joint commission of international accreditation	0.7906	6
Marketing effectiveness	Number of medical tourism agencies and facilitations	1.0052	5
Cost Advantage	Average price of medical procedures	0.0585	9

**Table 3:** Collected variables and situation of Grey relational generation.

Situation of Grey Relational Generation	Upper Bound Effectiveness Measurement								Lower Bound Effectiveness Measurement	Upper Bound Effectiveness Measurement
Thailand	35,592	6	4.5	0.8	221.92	2.1	68	8	11,808	600
Singapore	13,903	2	4.8	2.3	2462.39	2.4	20	2	12,690	150
Malaysia	25,948	2	4.5	1.5	361.52	1.9	13	5	8025	350
South Korea	13,336	6	4.8	3.7	2043.86	11.5	24	2	14,883	655
India	15,543	5	4.4	0.8	62.72	0.8	39	112	5783	450
Taiwan	10,740	1	4.3	2.2	2732	5.7	13	1	10,167	300

**Table 4:** Overall performance of six destinations of medical tourism in the Asia-Pacific region.

Medical Tourism Destinations	Grey Relational Grey Grade (Eigenvalues)	Ranking
Thailand	0.966152	1
Singapore	0.893754	4
Malaysia	0.944011	2
Taiwan	0.885301	6
India	0.915399	3
South Korea	0.893026	5

Note: Results of Grey relational analysis (GRA).

**Table 5:** Performance of proposed model.

Year	Annual Revenue (Billion NT$)	Relative Percentage Error (%)		Number of Medical Tourist (Person)	Relative Percentage Error (%)	
		GM (1,1) Model	Discrete Grey Model (DGM) (1,1) Model		GM (1,1) Model	DGM (1,1) Model
2012	9.623			173,311		
2013	13.648	0.35	0.52	231,164	1.02	0.51
2014	14.135	0.20	0.31	259,674	2.43	2.05
2015	15.896	7.86	7.81	305,045	8.03	7.78
2016	13.99	8.27	8.26	286,599	8.39	8.55
2017	14.727	6.37	6.30	305,618	12.55	12.58
2018	17.135	5.46	5.58	414,369	8.08	8.17
Mean absolute percentage error (MAPE, %)		4.75	4.80		6.75	6.61
Accuracy rate (%)		95.25%	95.2%		93.25%	93.39%
Predictive capability		Excellent	Excellent		Good	Good

**Table 6:** Estimated values of Taiwan’s medical tourism industry in the next six years.

Year	Results of the GM (1,1) Model		Results of the DGM (1,1) Model	
	Annual Revenue (Billion NT$)	Number of Medical Tourists (Persons)	Annual Revenue (Billion NT$)	Number of Medical Tourist (Person)
2019	16.75	421,779	16.72	420,827
2020	17.33	467,042	17.28	465,412
2021	17.92	517,163	17.86	514,721
2022	18.53	572,663	18.46	569,255
2023	19.16	634,118	19.08	629,566
2024	19.82	702,169	19.72	696,267
2025	20.50	777,523	20.38	770,034
